# Structural prediction of the interaction of the tumor suppressor p27^KIP1^ with cyclin A/CDK2 identifies a novel catalytically relevant determinant

**DOI:** 10.1186/s12859-016-1411-0

**Published:** 2017-01-05

**Authors:** Jinyu Li, Jörg Vervoorts, Paolo Carloni, Giulia Rossetti, Bernhard Lüscher

**Affiliations:** 1 College of Chemistry, Fuzhou University, Fuzhou, 350002 China; 2 Institute of Biochemistry and Molecular Biology, Medical School, RWTH Aachen University, 52057 Aachen, Germany; 3 Computational Biomedicine, Institute for Advanced Simulation IAS-5 and Institute of Neuroscience and Medicine INM-9, Forschungszentrum Jülich, 52425 Jülich, Germany; 4 Department of Oncology, Hematology and Stem Cell Transplantation, Medical School, RWTH Aachen University, Aachen, Germany; 5 Jülich Supercomputing Centre (JSC), Forschungszentrum Jülich, 52425 Jülich, Germany

**Keywords:** Cyclin-dependent kinase, Tumor suppressor, Molecular modeling, p27^KIP1^, Phosphorylation

## Abstract

**Background:**

The cyclin-dependent kinase 2 (CDK2) together with its cyclin E and A partners is a central regulator of cell growth and division. Deregulation of CDK2 activity is associated with diseases such as cancer. The analysis of substrates identified S/T-P-X-R/K/H as the CDK2 consensus sequence. The crystal structure of cyclin A/CDK2 with a short model peptide supports this sequence and identifies key interactions. However, CDKs use additional determinants to recognize substrates, including the RXL motif that is read by the cyclin subunits. We were interested to determine whether additional amino acids beyond the minimal consensus sequence of the well-studied substrate and tumor suppressor p27^KIP1^ were relevant for catalysis.

**Results:**

To address whether additional amino acids, close to the minimal consensus sequence, play a role in binding, we investigate the interaction of cyclin A/CDK2 with an in vivo cellular partner and CDK inhibitor p27^KIP1^. This protein is an intrinsically unfolded protein and, in particular, the C-terminal half of the protein has not been accessible to structural analysis. This part harbors the CDK2 phosphorylation site. We used bioinformatics tools, including MODELLER, iTASSER and HADDOCK, along with partial structural information to build a model of the C-terminal region of p27^KIP1^ with cyclin A/CDK2. This revealed novel interactions beyond the consensus sequence with a proline and a basic amino acid at the P + 1 and the P + 3 sites, respectively. We suggest that the lysine at P + 2 might regulate the reversible association of the second counter ion in the active site of CDK2. The arginine at P + 7 interacts with both cyclin A and CDK2 and is important for the catalytic turnover rate.

**Conclusion:**

Our modeling identifies additional amino acids in p27^KIP1^ beyond the consensus sequence that contribute to the efficiency of substrate phosphorylation.

## Background

Cell cycle progression is controlled by cyclin-dependent kinases (CDK) complexes. CDK2 in complex with E type cyclins regulates the transition through the restriction point in the G1 phase of the cell cycle and early events in S phase. A major function of cyclin A/CDK2 complexes is to promote DNA replication and the progression though S phase [1]. Consistent with these important functions are the involvement of CDK2 kinase complexes in diseases, including cancer [2]. Different cyclins are overexpressed while the expression of CDK inhibitors is reduced in different tumor entities [2]. These findings suggest that CDKs are promising targets for pharmaceutical interventions and CDK2 inhibitors are in clinical trials [3–5]. Moreover, Wee1, a tyrosine kinase repressor of CDK1 and CDK2, is targeted by small molecules to prevent repression of CDK1 and CDK2 in response to DNA damage and thus enhance genetic instability and apoptosis [6]. Thus the activation or repression of these CDKs is potentially beneficial to cancer patients dependent on the nature of the tumor [7]. Together, these findings suggest that the molecular understanding of the catalytic function of these kinases is both of fundamental and clinical relevance.

In general, cyclin/CDK2 complexes phosphorylate substrate proteins at S or T residues (the so-called P + 0 site, single amino acid code is used). Early on it was recognized that these kinases prefer S/T sites that are followed by a proline (at the P + 1 site) [8], an amino acid that is conserved across nearly all cyclin/CDK substrates. The use of oriented peptide libraries in kinase assays with cyclin A/CDK2 resulted in the description of HHASPRK as an optimal substrate peptide [9], with a basic residue frequently observed at the P + 3 site [9, 10]. This was used to obtain structural information of the cyclin A/CDK2 complex bound to a substrate peptide [11], supporting the conclusion that the amino acids at positions P + 0, P + 1 and P + 3 (underlined in HHASPRK) are recognized by the enzyme.

The above summarized findings relate to a rather small peptide. Whether such a sequence is sufficient for mediating phosphorylation in cells is not known. Frequently multiple lower affinity interactions contribute to functional specificity [12, 13]. One such motif for at least some cyclin/CDK complexes is the RXL sequence on substrates. Cyclin A can interact with this short sequence motif, which enhances specificity beyond the sequence recognized by the kinase domain, and was found to be important to mediate CDK2 phosphorylation [14]. Moreover, it is feasible that also the short sequence motif found to interact with the catalytic domain, which is characterized by a P at the P + 1 and a basic amino acid at the P + 3 site, is further expanded and thus might offer additional selectivity.

Defining interaction domains of substrates, beyond their minimal peptide sequence, with their enzymes allows more detailed molecular analysis. Of interest is to understand whether CDK2 recognizes its substrates by a common binding mode beyond the consensus sequence S/T-P-X-R/K/H described above. Thus, do longer peptide chains, as present in real proteins, affect binding and phosphorylation? To know more about the interaction of CDK2 with a substrate is also indicated as CDK2 inhibitors are evaluated as therapeutic tools in clinical trials [7]. More detailed knowledge of enzyme-substrate interaction may allow defining more selective inhibitors beyond ATP analogs. Here we address this issue by using homology modeling, docking, and bioinformatics conservation analyses and biochemical methods. We focus on the tumor suppressor p27^KIP1^, whose cyclin E/CDK2 and cyclin A/CDK2-dependent phosphorylation results in subsequent degradation [15, 16]. We observe that additional interactions of p27^KIP1^ with CDK2 are relevant to enhance phosphorylation rates.

## Results and Discussion

### Consensus sequences of CDK2 substrates

**Fig. 1:**
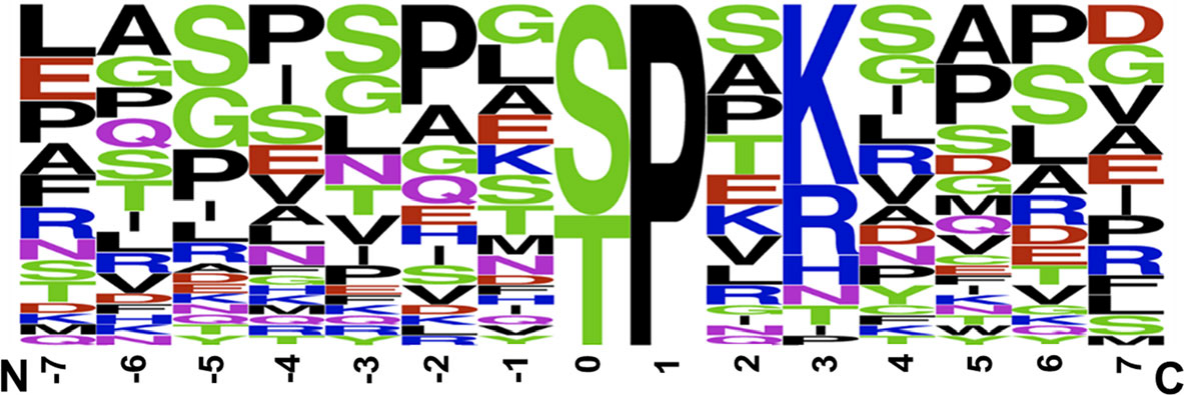
Sequence logo plot represent normalized amino acid frequencies for ±7 amino acids from the phosphorylation site of CDK2 in vivo substrates reported in Table 1. This figure was generated using WebLogo [76]

The majority of information on substrate sequences for CDK2 has been obtained from analyzing peptide and protein substrates using in vitro kinase assays. We wanted to define whether the CDK2 consensus substrate sequence extends beyond the S/T-P-X-R/K/H motif when sites that were confirmed in cells were used. We used PhosphoSitePlus [17] to define potential CDK2 substrates. We then screened the relevant publications i) for direct phosphorylation of the substrate by cyclin/CDK2 including mapping of the site phosphorylated in vitro, ii) for phosphorylation of this site in cells using mass spectrometry and/or phosphospecific antibodies, and iii) for altered phosphorylation of this site in response to modulating CDK2 activity by genetic means or for phosphorylation in a cell cycle-dependent manner that is consistent with CDK2 activity. Thirty-five CDK2 sites of a total of 27 substrates fulfilled these criteria (Table 1). Sequence logo analysis of these CDK2 sites suggested that the S/T-P motif is invariable and that nearly 80% of the substrates possess a basic amino acid at P + 3 (Fig. 1). Thus, most substrates fulfill the requirements of the consensus sequence obtained by using peptide substrates. Little preference for other amino acids at any other position was detected. This is consistent with the concept that additional determinants of various nature, including the RXL motif [14], might contribute to the substrate specificity in cells [13]. Moreover, additional amino acids in the sequence that is modified and that is recognized near the catalytic cleft may contribute to selectivity. Together several different weak determinants may contribute to the overall affinity of an individual substrate and thus contribute to the efficiency of phosphorylation.

**Table 1:** Information on human CDK2 substrates confirmed by in vivo experiments

Index	Protein	UniProtKB	P + 0 site	Site sequence	Ref.
1	ATRIP	Q8WXE1	S224	VSHVSPRKNPSV	[50]
2	BRCA1	P38398	S1497	VERSSPSKCPSL	[51]
3	CDKN2D	P55273	S76	SGTSPVHDAAR	[52]
4	CROCC iso2	Q5TZA2-2	S763	PVPGSPARDAPA	[53]
5	Ctip	Q99708	T847	IPPNTPENFWEV	[54]
6	DLG1	Q12959	S158	HSHISPIKPTEA	[55]
7		Q12959	S443	QTPASPARYSPV	
8	EZH2	Q15910	T345	ERIKTPPKRPGG	[56]
9	FOXM1 iso2	Q08050-2	T596	PISSTPSKSVLP	[57]
10	FOXO1A	Q12778	S249	KSGKSPRRRAAS	[58]
11	HIRA	P54198	T555	SVLTTPSKIEPM	[59]
12	HR6A	P49459	S120	PNPNSPANSQAA	[60]
13	ID3	Q02535	S5	MKALSPVRGCYE	[61]
14	ING5	Q8WYH8	T152	EEEDTPKKKKHK	[62]
15	LIG3	P49916	S210	GQVTSPVKGASF	[63]
16	ORC2	Q13416	T116	ELAKTPQKSVSF	[64]
17		Q13416	T226	VGKETPSKRMKR	
18	p27	P46527	T187	SVEQTPKKPGLR	[65]
19	p63	Q9H3D4	T491	RNALTPTTIPDG	[66]
20	p73	O15350	T86	ASPYTPEHAASV	[67]
21	PELP1	Q8IZL8	S991	PPPESPPKVQPE	[68]
22	PLZF	Q05516	S197	LSAMSPTKAAVD	[69]
23		Q05516	T282	EGPGTPTRSSVI	
24	PTPN2 iso2	P17706-2	S304	AFDHSPNKIMTE	[70]
25	RbBP1	P29374	S864	LGQSSPEKKIRI	[71]
26		P29374	S1007	FSVASPLTLSQD	
27	RPL12	P30050	S38	PLGLSPKKVGDD	[72]
28	SIRT2	Q8IXJ6	S368	STSASPKKSPPP	[49]
29	Smad3	P84022	T8	ILPFTPPIVKRL	[73]
30		P84022	T179	NIPETPPPGYLS	
31		P84022	S213	PNPMSPAHNNLD	
32	SMRT	Q9T618	S1259	IGEDSPSRLDRG	[74]
33		Q9T618	T1463	ITQGTPLKYDTG	
34		Q9T618	S1487	SLIGSPGRTFPP	
35	NBS1	Q9NX02	S432	NYQLSPTKLPSI	[75]

### Structural model of a p27^KIP1^ substrate peptide with cyclin A/CDK2

**Fig. 2:**
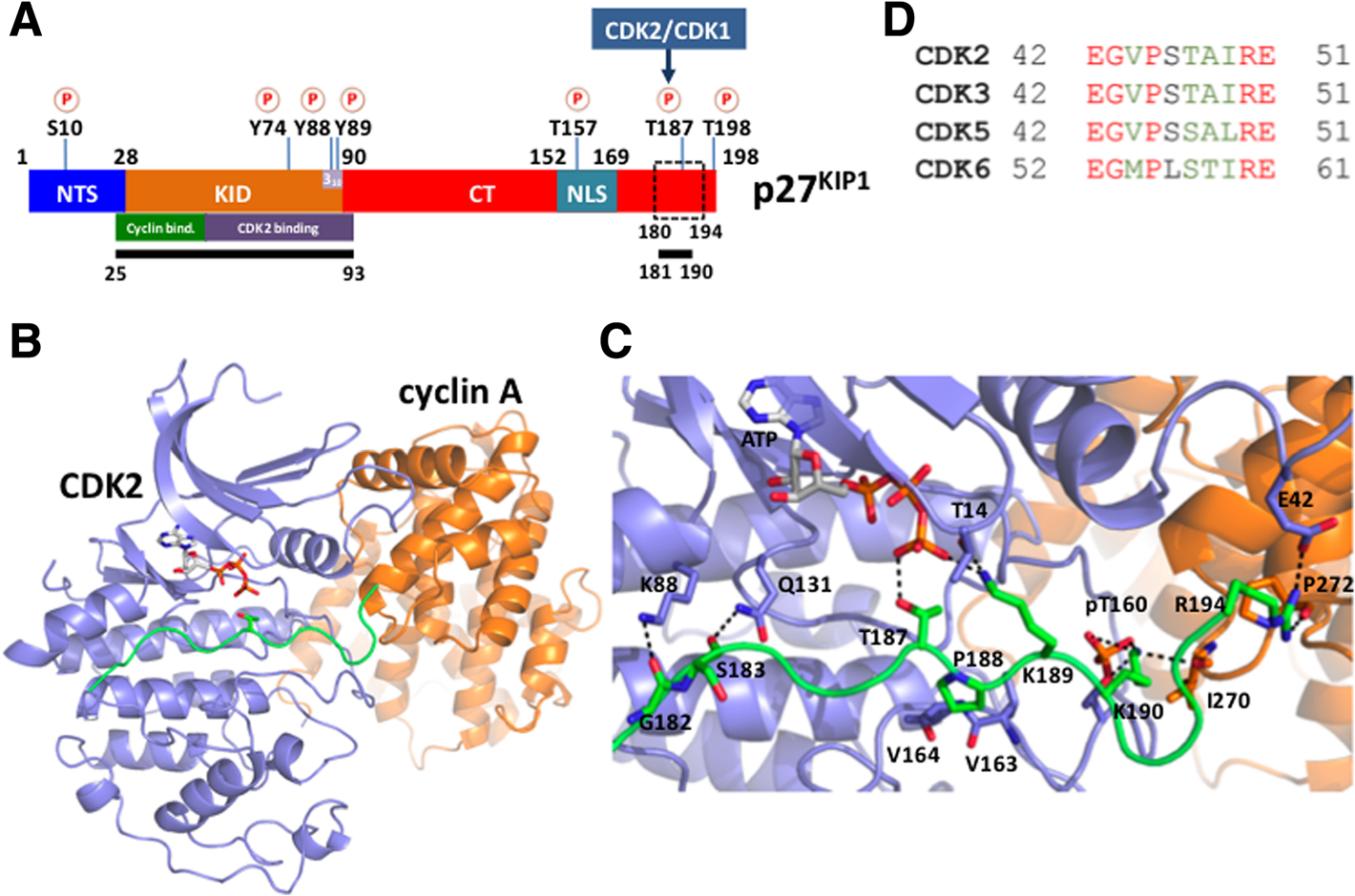
Schematic visualization of p27^KIP1^ and structural model of the cyclin A/CDK2/p27^KIP1^ peptide (amino acids 180–194) complex. **a** Schematic representation of the domain organization of p27^KIP1^. The N-terminal segment (NTS, residues 1–28), the N-terminal kinase inhibitory domain (KID, residues 28–90), the C-terminal region (CT, residues 91–198), and the nuclear localization sequence (NLS, residues 152–169) are indicated. The known phosphorylation sites observed in p27^KIP1^ are also indicated [15]. The cyclin and CDK2 binding regions in KID are highlighted [21]. The available crystal structure data for p27^KIP1^ are highlighted by black bars (PDB ID: 1JSU (residues 25–93) [21] and 2AST (residues 181–190) [29]). The C-terminal binding region (residues 180–194), for which a structural model was constructed, is indicated by a dashed box. **b** The overall structure is shown in cartoon representation. CDK2, cyclin A, and the peptide substrate (amino acids 180–194 of p27^KIP1^) are colored in blue, orange, and green, respectively. The model contains ATP. **c** Close-up view of the binding interface between p27^KIP1^ and cyclin A/CDK2. Hydrogen bonds are highlighted in black dashed lines. **d** Sequence alignment showing the conservation of E42 in CDK2 compared with other CDKs. The sequence alignment was obtained using the Clustal Omega webserver [42]. Identical and structurally similar residues are indicated in red and green, respectively

We have addressed the role of additional amino acids close to the consensus sequence for the tumor suppressor p27^KIP1^, one of the best-studied substrates of CDK2 complexes. p27^KIP1^ functions as an inhibitor of CDK2 and CDK1 complexes and thus has the ability to interfere with cell cycle progression, for example in response to anti-proliferative signals [16]. p27^KIP1^ is a naturally unfolded protein that is largely disordered in solution [18–20]. Thus, determining its conformation based on computational methods is currently very challenging. In complex with cyclin A/CDK2, the N-terminal portion of p27^KIP1^ (amino acids 25–93) assumes a stable conformation. In the co-crystal of cyclin A/CDK2—p27^KIP1^ two binding regions in the latter were identified [21]. Amino acids 25–50 of p27^KIP1^ interact with cyclin A, whereas amino acids 52–93 bind to CDK2. This second interaction region includes the so-called 3_10_ helix, which contains Y88 (for a structure-function comparison of p27^KIP1^ see Fig. 2a). Y88 is substrate of non-receptor tyrosine kinases [22]. Y88 phosphorylation triggers a conformational transition and ejects the p27^KIP1^ 3_10_ helix from the CDK2 catalytic cleft [23, 24]. This in turn allows the binding of the C-terminal part of p27^KIP1^ and the subsequent phosphorylation of T187 by CDK2 [22]. This site is part of the CDK2 consensus sequence with T_187_PKK in p27^KIP1^.

No structural information is presently available for the C-terminal half of p27^KIP1^ (residues 91–198). There are two main strategies for structural predictions of protein complexes [25]. Template-based docking is a high-throughput method, which performs fairly well when using with high- and medium-sequence or structural similarity (root-mean-square deviation, RMSD <6 Å) between template and target. This is not the case for CDK2 substrates as the peptide (HHASPRK) in the only available crystal structure of a cyclin A/CDK2/substrate complex is not large enough to provide sufficient information of the protein-protein interface for template-based docking [11]. Because of this, free docking is the method of choice with the interacting proteins using the available experimental constraints. As pointed out above, p27^KIP1^ is disordered and no structural information is available, specifically of the C-terminal half of the protein that contains the cyclin A/CDK2 phosphorylation site. The occurrence of disordered regions for kinase substrates is common, because kinases normally phosphorylate sites in less ordered regions that are exposed on the surface of proteins [26–28]. Hence, we applied homology modeling, along with existing information of identical short amino acid sequences with known structures, to construct structural models of the interactor [11, 29]. The docking was guided by two key observations. First, the phosphorylation site of the substrate forms an H-bond with ATP [11]. Second, the basic residue at the P + 3 site forms H-bonds to the phosphorylated T160 (pT160) of CDK2 and I270 of cyclin A [11]. Phosphorylation of T160 in the activation loop is critical for catalytic activity of CDK2 [30]. Consistent with a role of this kinase in late G1, S and G2, phosphorylation of T160 is high in these phases of the cell cycle [31].

**Fig. 3:**
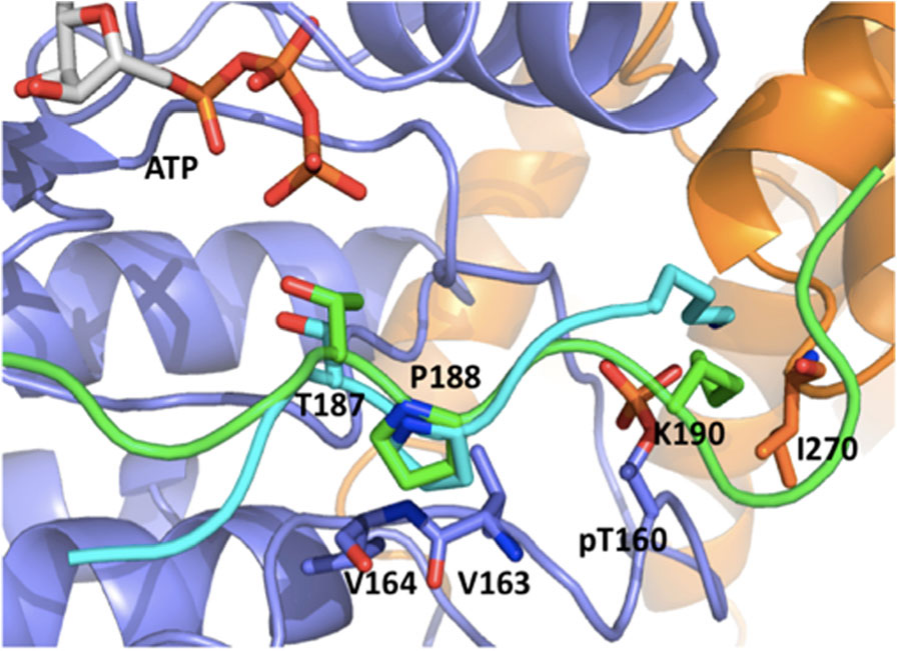
Superposition of the predicted model of cyclin A/CDK2/p27^KIP1^ with cyclin A/CDK2/HHASPRK X-ray structure (PDB ID: 1QMZ [11]). The cartoon representations of CDK2, cyclin A, and the p27^KIP1^ peptide (amino acids 180–194) are colored in blue, orange, and green, respectively. The peptide substrate in the crystal structure is colored in cyan

The p27^KIP1^ peptide (amino acids 180 to 194) adopted an extended conformation in the active site of CDK2 (Fig. 2b and c). The interactions that were noticed between CDK2 and the substrate in the crystalized cyclin A/CDK2/HHASPRK structure [11] and in the modeled cyclin A/CDK2/SIRT2 complex [32] are maintained. The positions of T187 and P188 are very similar in our model to those in the cyclin A/CDK2/HHASPRK structure (Fig. 3). The T187, the P + 0 site amino acid, interacts with ATP through an H-bond. More C-terminal the structures start to deviate clearly seen with K190, which moves considerably due to the additional amino acids in the p27^KIP1^ peptide (amino acids 180 to 194) but still interacts with pT160 of CDK2 and I270 of cyclin A (Figs. 2c and 3).

### Lysine at the P + 2 site and turnover rate

**Fig. 4:**
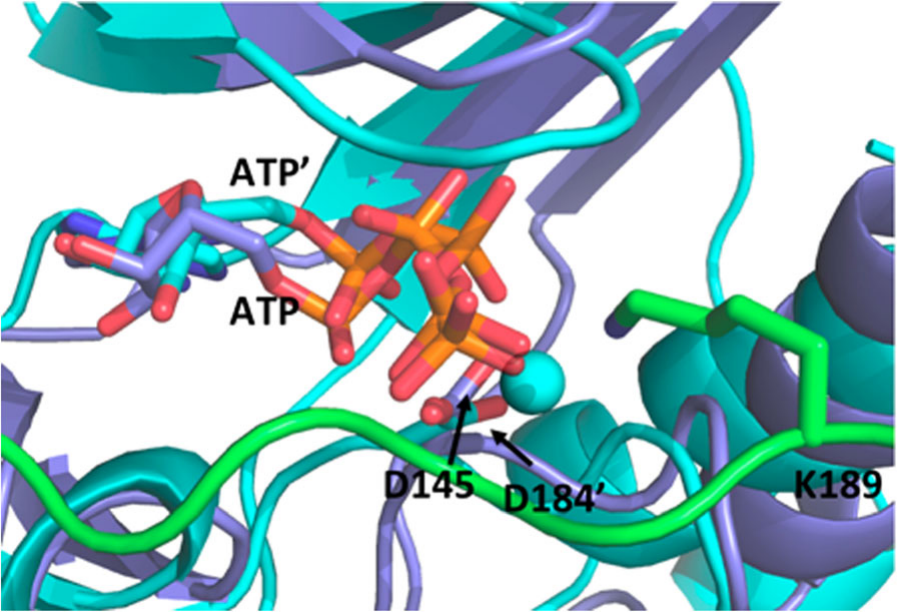
Superposition of cyclin A/CDK2/p27^KIP1^ peptide (amino acids 180–194) with the PKA/ATP/2 Mn^2+^ crystal structure (PDB ID: 1AST [36]). The second Mn^2+^ in PKA is indicated by cyan sphere. The cartoon representations of CDK2 and p27^KIP1^ are colored in blue and green, respectively. PKA is colored in cyan. PKA residues are labeled with prime

In contrast to the crystal structure [11], in which the arginine at the non-conserved P + 2 site does not have any contact with cyclin A/CDK2, the lysine at the P + 2 site of p27^KIP1^ (K189) forms a salt bridge with ATP and an H-bond with T14 of CDK2 (Fig. 2c). The first cyclin A/CDK2 structure solved contained one metal ion as part of the ATP · Mg^2+^ cofactor complex [11, 33], while many other kinases contain two. In a more recent study, a cyclin A/CDK2 transition state complex with ADP, MgF_3_^–^ (a mimic for the γ-phosphate in the transition state), and a short peptide was crystallized, which revealed a second Mg^2+^ ion [34]. Based on this structure, a mechanistic analysis suggested that CDK2 also uses 2 Mg^2+^ ions [35]. The second Mg^2+^ ion appears to associate reversibly with the active site. It is required just before the chemical step of the catalytic cycle and dissociates as soon as the reaction is complete. Thus, it promotes efficient phosphoryl transfer. But this second Mg^2+^ ion also increases, together with the other Mg^2+^ ion, the affinity of ADP and it therefore prevents efficient release. This would reduce the overall turnover rate of the enzymatic reaction [35]. Keeping this in mind, we compared our model with the crystal structure of protein kinase A (PKA) [36]. We observed that the location of the positively charged nitrogen atom of K189 of p27^KIP1^ is similar to that of the second metal ion in PKA (Fig. 4). We speculate that K189 and its positive charge may affect the stability and/or the positioning of the second Mg^2+^ ion and facilitate its release once the phosphoryl transfer is completed. In consequence this may result in an acceleration of the turnover rate. In support of this model, short CDK2 substrate peptides with K or R at the P + 2 position show the highest velocity of phosphoryl transfer, while acidic amino acids reduce velocity considerably [10]. Together these findings indicate that the second Mg^2+^ ion promotes catalysis and in combination with a basic residue at the P + 2 site of the substrates the price for a reduced turnover rate does not have to be paid [37].

### Specific contacts of the P + 7 arginine of the substrate peptide with cyclin A/CDK2

Additional contacts were identified in our model of the trimeric cyclin A/CDK2/p27^KIP1^ peptide (amino acids 180-194) complex between the non-consensus part of p27^KIP1^ and cyclin A/CDK2. S183 and G182 of the p27^KIP1^ peptide make H-bonds with the active site residues Q131 and K88 of CDK2, respectively (Fig. 2c). Importantly, R194 of p27^KIP1^ forms H-bonds with E42 of CDK2 and P272 of cyclin A, both of which are located at the binding interface between CDK2 and cyclin A. This glutamate is conserved across CDK2, CDK3, CDK5 and CDK6 and belongs to the extension loop of the PSTAIRE helix of CDK2 (residues 45–51) (Fig. 2d), which shows the most significant movement upon cyclin A binding [33]. Thus, R194 may play a role in the phosphorylation of p27^KIP1^ by cyclin A/CDK2 through interacting with and altering the position of the extended PSTAIRE loop. This might stabilize the active cyclin A/CDK2 complex.

### Arginine at the P + 7 position enhances the enzymatic turnover rate

**Fig. 5:**
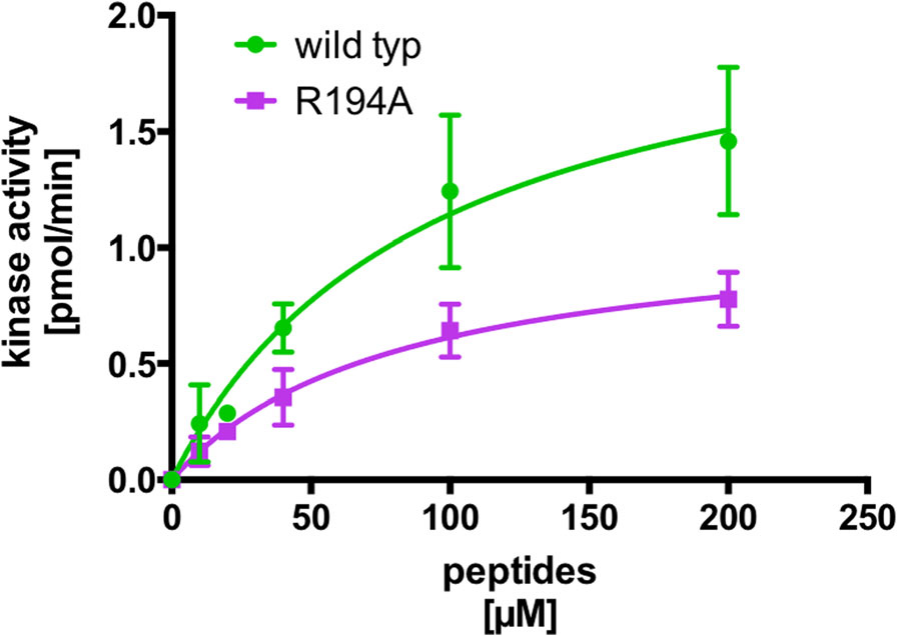
Analysis of cyclin A/CDK2 kinase activity on p27^KIP1^ substrate peptides. Cyclin A/CDK2 Kinase purified from Sf9 cells was incubated with increasing concentrations of p27^KIP1^ wild type and the R194A mutant peptide as indicated. Phosphorylation of the peptides was monitored after immobilisation on SAM2 membrane by scintillation counting and analysis by GraphPad Prism software. Shown are mean values ± SD of 5 experiments performed in duplicates

To validate our model, we performed in vitro kinase assays. A p27^KIP1^ wild-type (wt) peptide (amino acids 180 to 194) and a peptide with an R194A substitution were synthesized and their capacity to be phosphorylated by cyclin A/CDK2 was evaluated. We determined the kinetic constants for these synthetic peptides. The K_M_ values of the two peptides were very similar with 93.6 and 81.4 μM for the wt and R194A, respectively (Fig. 5 and Table 2). However, we observed a difference in the maximal relative rate of phosphoryl transfer between the two peptides, i.e. 2.21 and 1.11 pmol/min for the wt and the R194A peptide, respectively (Fig. 5 and Table 2). Thus the turnover rate of the wt peptide was twice as high as of the R194A mutant peptide. It is possible that the interaction of R194 with both cyclin A and CDK2 allows a faster correct orientation of the P + 0 site amino acid T187 in the catalytic cleft. This data suggests that beside the core consensus sequence defined by the HHASPRK peptide additional residues can be involved in determining the efficiency of substrate phosphorylation.

**Table 2:** Kinetic constants for phosphorylation of p27^KIP1^ peptides

Peptide	Sequence	K_m_ [μM]	Relative rate [pmol/min]	Relative rate/K_m_
wt	N_180_AGSVEQTPKKPGLR	93.6	2.21	0.0195
R194A	N_180_AGSVEQTPKKPGLA	81.4	1.11	0.0135

## Conclusions

The minimal consensus sequence for CDK2 and several other CDKs matches the sequence S/T-P-R/K/H. This information is mainly drawn from work with short model peptides as substrates [9–11]. To increase specificity of an enzyme-substrate interaction, additional determinants are required, which may be distinct between substrates. In more general terms it has been argued that during evolution multiple lower affinity interactions have been selected to contribute to functional specificity [12, 13]. Intuitively, one might expect that systems develop towards high affinity interactions. However, this seems not to be the case. In fact, signaling, and thus the flow of information, often involves non-structured parts of proteins and low affinity interaction. Nevertheless, these processes are remarkably specific and functional. Moreover, proximity is another important aspect to create specificity [38]. This may include the use of scaffold proteins, targeting subunits or specific microenvironments that allow the co-localization of proteins. All these aspects contribute to the substrate specificity of enzymatic reactions. For example the use of scaffold proteins has been well documented for the activation of MAP kinases in different signaling pathways [39]. In some respect cyclins also have scaffolding function, in addition to activating the CDK. For some cyclin/CDK complex substrates an RXL motif, which is recognized by the cyclin, is important for efficient phosphorylation [40]. Here we identified amino acid P + 7 of the CDK2 phosphorylation site at T187 in p27^KIP1^ as important for the phosphoryl transfer rate. R194 enhances the turnover rate of a peptide substrate compared to a mutant peptide with R194A. The P + 7 site is not conserved in cellular CDK2 substrates, suggesting that it has a unique effect on p27^KIP1^ as a key substrate in the control of cell cycle progression. In other substrates, other changes may affect the enzymatic properties and thus, depending on the specific needs, different alterations in substrates may contribute to the efficiency of phosphorylation by CDK2. Moreover, the identification of the amino acid P + 7 of the substrate as relevant for catalysis suggests that determinants relatively far from the site of modification could be relevant for interfering with CDK2 activity. Such determinants could contribute to developing more selective inhibitors, which might be relevant in light of the interest in developing selective CDK inhibitors for therapeutic purposes [7].

## Methods

### Modeling of cyclin A/CDK2/p27^KIP1^ complexes

**Fig. 6:**
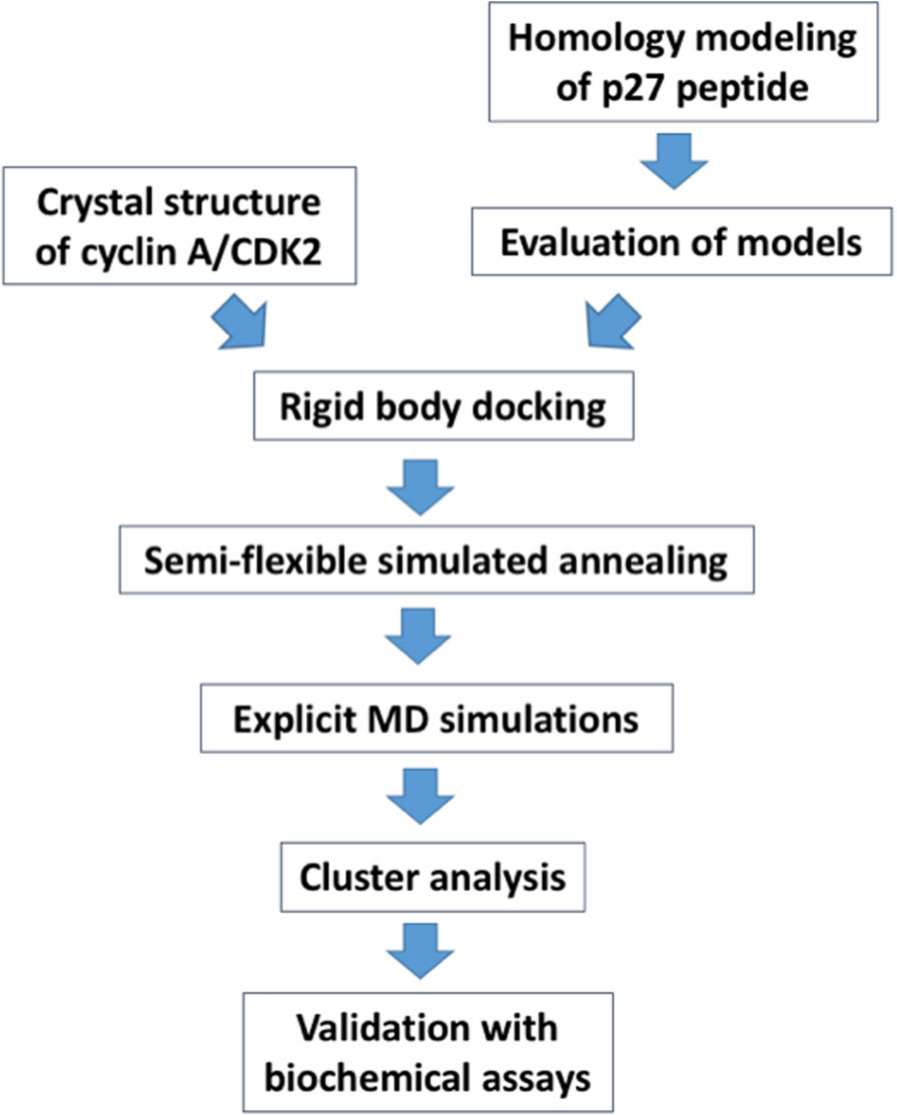
Flow chart describing the modeling process as detailed in the Methods section

Structural predictions of p27^KIP1^ is challenging because the protein is largely disordered. In particular no information is available for the C-terminal half of the protein. We constructed models of the C-terminal p27^KIP1^ peptide (residues 180–194, sequence N_180_AGSVEQTPKKPGLR, see Fig. 2a) based on the X-ray structure of the p27^KIP1^ peptide A_181_GSVEQTPKK [29] and the 30S ribosomal protein S8 peptide K_81_PGLR [41], of which the sequence identities to the p27^KIP1^ peptide are 67% and 33%, respectively (for a flow chart of the procedure, see Fig. 6). 200 models of the p27^KIP1^ C-terminal peptide were generated using the automodel class implemented in MODELLER 9v9 package [42]. Sixty-four models turned out to have 90% or more of the amino acids in the most favored regions of the Ramachandran plot [43]. We selected the model that showed the lowest difference regarding backbone RMSD (1.1 Å) with respect to the C-terminal peptide 180–194 and the crystal structure of amino acids 181–190 of p27^KIP1^ [29]. This model was also in agreement with an analysis using the iTASSER server [44], from which we obtained five models. All were in an extended conformation similar to the results obtained with MODELLER 9v9 [42] (i.e. backbone RMSD between 0.15 to 0.22 nm, data not shown). The selected model was further evaluated by using MolProbity server [45]. The MolProbity score of the model is 97th percentile closing to 100th percentile of the best structure in the dataset, which shows a good quality of the model. The structure of cyclin A/CDK2 in complex with ATP · Mg^2+^ was obtained from a previous study [11]. Data-driven docking calculations of cyclin A/CDK2 with the p27^KIP1^ peptide was carried out as described [32]. The model of cyclin A/CDK2 is based on the X-ray structure of human CDK2 in complex with cyclin A and the HHASPRK peptide (PDB ID: 1QMZ [11]). This contains one Mg^2+^ ion bound to a water molecule, the ATP cofactor, and N132 and D145 of CDK2. In the docking calculations, we considered only non-hydrogen atoms and imposed restraints between the phosphorylation site of the substrate and ATP, because the contact between ATP and the phosphorylation site is the prerequisite for the phosphoryl group transfer reaction [46], and between the basic residue at the P + 3 site of the substrate, pT160 of CDK2, and I270 of cyclin A, because these contacts are crucial for substrate specificity, as indicated by the crystal structure of cyclin A/CDK2•HHASPRK [11]. Distance restraints were applied on Mg^2+^ ions and their ligands to preserve the coordination structure using the HADDOCK program [47, 48]. Rigid body docking, semi-flexible simulated annealing and explicit molecular dynamics refinement implemented in HADDOCK [47, 48] were carried out with the default parameters. Hydrogen bonds were defined to be present if the distance between the acceptor and donor atoms is below 3.5 Å and the angle among the hydrogen-donor-acceptor atoms are below 30°. All figures for the visualization of structures were drawn using PyMOL (Molecular Graphics System, Version 1.3, Schrödinger LLC).

### Protein purification and in vitro kinase assay

Human cyclin A/CDK2 complexes were expressed in SF9 insect cells (*Spodoptera frugiperda*) upon infection with recombinant baculo viruses encoding either cyclin A or CDK2. The complex was purified using the GST tag of cyclin A [49]. In vitro kinase assays of cyclin A/CDK2 were performed in kinase buffer (50 mM Tris-HCl, pH 7.5, 10 mM MgCl_2_, 1 mM EGTA, 1 mM DTT, 40 mM α-glycero-phosphate, 20 mM *p*-nitrophenylphosphate, 0.1 mM sodium vanadate, 0.01% BriJ 35) containing 50 μM ATP and 1 μCi ^32^P-γ-ATP for 10 min at 30°C in the presence of increasing amount of biotinylated p27^KIP1^ wild type or R194A (an arginine to alanine substitution at position 194) peptides as substrates. The assays were terminated by adding 12.5 μl 7.5 M guanidine hydrochloride. The peptides were immobilized on SAM2 Biotin Capture Membranes (Promega), extensively washed as described in the manufacturer’s protocol, and then monitored for radioactivity in a liquid scintillation counter to calculate the specific kinase activity. Kinetic parameters were determined with GraphPad Prism software built-in “Enzyme kinetic Michaelis-Menten”. The sequence of the p27^KIP1^ wild type peptide corresponds to amino acids 180 to 194. The peptides were synthesized with a biotin-PEG-20-linker and purified by HPLC at the Peptide Specialty Laboratories, Heidelberg (Germany).

## Abbreviations

ATP: Adenosine triphosphate
CDK2: Cyclin-dependent kinase 2
CT: C-terminal region
DTT: Dithiothreitol
GST: Glutathione S-transferase
KID: Kinase inhibitory domain
NLS: Nuclear localization sequence
NTS: N-terminal segment
PDB: Protein data bank
PKA: Protein kinase A
pT: Phosphorylated threonine
RMSD: Root-mean-square deviation
